# 3-Benzyl-6-(2-chloro­benzo­yl)-1,3-benzoxazol-2(3*H*)-one

**DOI:** 10.1107/S1600536810046301

**Published:** 2010-11-17

**Authors:** Yuldash R. Takhirov, Dilshod A. Dushamov, Kambarali K. Turgunov, Nasirkhon S. Mukhamedov, Khusniddin M. Shakhidoyatov

**Affiliations:** a Urgench State University named after Al-Khorezmiy, Kh. Olimjon Str. 14, Urgench 220100, Uzbekistan; b S. Yunusov Institute of the Chemistry of Plant Substances, Academy of Sciences of Uzbekistan, Mirzo Ulugbek Str. 77, Tashkent 100170, Uzbekistan

## Abstract

In the title compound, C_21_H_14_ClNO_3_, the benzoxazolone ring system is planar (r.m.s. deviation = 0.022 Å) and forms dihedral angles of 75.38 (10) and 65.92 (13)° with the mean planes of the chloro­benzoyl (r.m.s. deviation = 0.045 Å, excluding O atom) and benzyl (r.m.s. deviation = 0.023 Å) groups. The observed structure is stabilized by weak C—H⋯O hydrogen bonds and weak inter­molecular C—H⋯π inter­actions.

## Related literature

For the natural source of benzoxazolin-2-one and its derivatives, see: Tang *et al.* (1975 ▶); Chen & Chen (1976 ▶); Smissman *et al.* (1957 ▶). For the synthesis of benzoxazolin-2-one derivatives, see: Honkanen & Virtanen (1961 ▶); Bredenberg *et al.* (1962 ▶); Mukhamedov *et al.* (1994 ▶). For related structures, see: Groth (1973 ▶); Işık *et al.* (2004 ▶). For bond-length data, see: Allen *et al.* (1987 ▶).
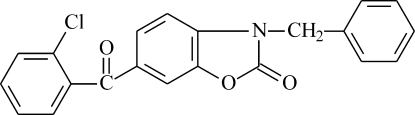


## Experimental

### 

#### Crystal data

C_21_H_14_ClNO_3_
*M* *_r_* = 363.78
Monoclinic, 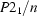

*a* = 13.391 (7) Å
*b* = 7.317 (6) Å
*c* = 18.611 (9) Å
β = 109.72 (4)°
*V* = 1716.6 (19) Å^3^
*Z* = 4
Mo *K*α radiation
μ = 0.24 mm^−1^
*T* = 293 K
0.80 × 0.40 × 0.07 mm

#### Data collection

Stoe Stadi-4 four-circle diffractometer
3452 measured reflections
2987 independent reflections
1866 reflections with *I* > 2σ(*I*)
*R*_int_ = 0.099
3 standard reflections every 60 min  intensity decay: 3.7%

#### Refinement

*R*[*F* ^2^ > 2σ(*F* ^2^)] = 0.072
*wR*(*F* ^2^) = 0.205
*S* = 1.06
2987 reflections
235 parameters
H-atom parameters constrained
Δρ_max_ = 0.28 e Å^−3^
Δρ_min_ = −0.32 e Å^−3^

### 

Data collection: *STADI4* (Stoe & Cie, 1997 ▶); cell refinement: *STADI4*; data reduction: *X-RED* (Stoe & Cie, 1997 ▶); program(s) used to solve structure: *SHELXS97* (Sheldrick, 2008 ▶); program(s) used to refine structure: *SHELXL97* (Sheldrick, 2008 ▶); molecular graphics: *XP* (Bruker, 1998 ▶); software used to prepare material for publication: *SHELXL97*.

## Supplementary Material

Crystal structure: contains datablocks I, global. DOI: 10.1107/S1600536810046301/jj2063sup1.cif

Structure factors: contains datablocks I. DOI: 10.1107/S1600536810046301/jj2063Isup2.hkl

Additional supplementary materials:  crystallographic information; 3D view; checkCIF report

## Figures and Tables

**Table 1:** Hydrogen-bond geometry (Å, °) *Cg*1 is the centroid of the C16–C21 ring.

*D*—H⋯*A*	*D*—H	H⋯*A*	*D*⋯*A*	*D*—H⋯*A*
C14—H14*A*⋯O2^i^	0.93	2.59	3.266 (8)	130
C20—H20*A*⋯O3^i^	0.93	2.59	3.269 (8)	130
C11—H11*A*⋯*Cg*1^ii^	0.93	2.92	3.474 (7)	119

Symmetry codes: (i) 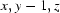; (ii) 
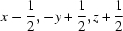
.

**Fig. 1.**
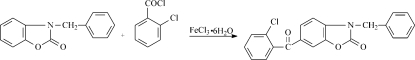
The reaction scheme for (I).

**Fig. 2.**
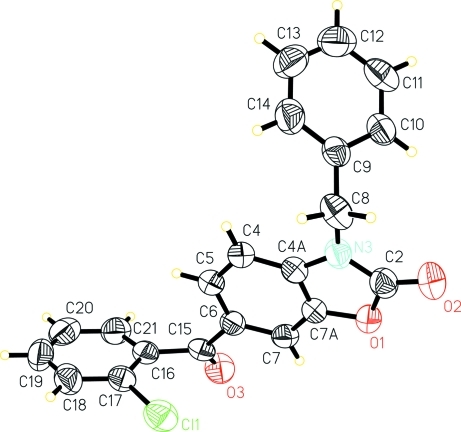
The molecular structure of the title compound,(I), with atom labels and 50% probability displacement ellipsoids for non-H atoms.

**Fig. 3.**
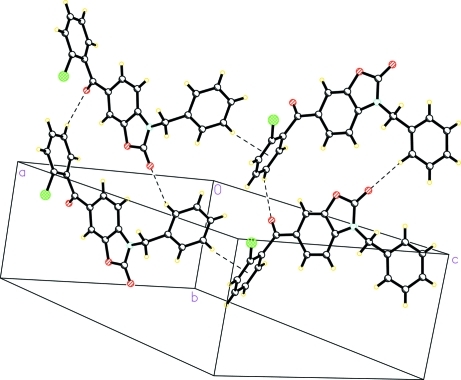
Packing diagram of the title compound, showing weak C—H···O hydrogen bonds and weak C–H···π-ring intermolecular interactions (dashed lines).

## supplementary crystallographic information

### Comment

Benzoxazolin-2-one and its derivatives were found in rye seedlings, roots of *Coix Lacryma Jobi L.* and *Scoporia dulcus* and possess physiological activity (Tang *et al.*, 1975; Chen & Chen, 1976; Smissman *et al.*, 1957).  Acylation of benzoxazolin-2-ones using FeCl_3_^.^6H_2_O as a catalyst, in low yields, has been demonstrated (Mukhamedov *et al.*, 1994).  Our efforts toward acylation of benzoxazolin-2-one derivatives, containing an additional aromatic ring, has led to the synthesis of the title compound, (I), C_21_H_14_ClNO_3_.

In the title compound,(I), the benzoxazolone ring system is planar with an r.m.s. deviation of 0.022 Å.  The dihedral angles between the mean planes of the benzoxazolone ring system and benzyl plane (r.m.s.deviation of 0.023Å) is 65.92 (13)° (Fig. 2).  The carbonyl group is twisted by 61.6 (3)° relative to the mean plane of the chlorophenyl group.  The dihedral angle between the benzoxazolone ring system and chlorophenyl plane (r.m.s. deviation of 0.045 Å) is 75.38 (10)°.  Bond distances and angles are in normal ranges (Allen *et al.*, 1987).  The observed structure is stabilized by weak C—H···O hydrogen bonds (Table 1).  In addition, weak C–H···π-ring intermolecular interactions are also observed (Fig. 3) [H11A···Cg1^ii^ = 2.92Å; C11···Cg1^ii^ = 3.474 (7)Å; C11—H11A···Cg1^ii^ = 119°; where Cg1 = C16–C21; ^ii^ = -1/2 +*x*, 1/2 - *y*, 1/2 + *z*].

### Experimental

To a powder of 3-benzylbenzoxazolin-2-one (2.25 g, 10 mmol) was added 2-chloro-benzoylchloride (2.625 g, 1.899 ml, d=1.382 g/ml, 15 mmol) and FeCl_3_.6H_2_O (0,027 g, 0.1 mmol) as a catalyst (Fig. 1). The reaction mixture was heated to 423–433 K for 4 h. After cooling, the product was washed with water and re-crystallized from ethanol. The title compound with m.p. 401–403 K was obtained in a yield of 80% (3.2 g). Crystals suitable for X-ray analysis were obtained from ethanol by slow evaporation.

### Refinement

Carbon-bound H atoms were positioned geometrically and treated as riding on their C atoms, with C—H distances of 0.93 Å (aromatic) and 0.97 Å (CH_2_) and were refined with *U*_iso_(H) =1.2*U*_eq_(C). All other non-H atoms were refined anisotropically.

### Crystal data

**Table:**

C_21_H_14_ClNO_3_	*F*(000) = 752
*M**_r_* = 363.78	*D*_x_ = 1.408 Mg m^−^^3^
Monoclinic, *P*2_1_/*n*	Melting point: 401(2) K
Hall symbol: -P 2yn	Mo *K*α radiation, λ = 0.71073 Å
*a* = 13.391 (7) Å	Cell parameters from 32 reflections
*b* = 7.317 (6) Å	θ = 5–15°
*c* = 18.611 (9) Å	µ = 0.24 mm^−^^1^
β = 109.72 (4)°	*T* = 293 K
*V* = 1716.6 (19) Å^3^	Plate, colourless
*Z* = 4	0.80 × 0.40 × 0.07 mm

### Data collection

**Table:**

Stoe Stadi-4 four-circle diffractometer	*R*_int_ = 0.099
Radiation source: fine-focus sealed tube	θ_max_ = 25.0°, θ_min_ = 1.6°
graphite	*h* = −15→14
Scan width (ω) = 0.90 – 1.71, scan ratio 2θ:ω = 1.00 I(Net) and sigma(I) calculated according to Blessing (1987)	*k* = 0→8
3452 measured reflections	*l* = 0→22
2987 independent reflections	3 standard reflections every 60 min
1866 reflections with *I* > 2σ(*I*)	intensity decay: 3.7%

### Refinement

**Table:**

Refinement on *F*^2^	Primary atom site location: structure-invariant direct methods
Least-squares matrix: full	Secondary atom site location: difference Fourier map
*R*[*F*^2^ > 2σ(*F*^2^)] = 0.072	Hydrogen site location: inferred from neighbouring sites
*wR*(*F*^2^) = 0.205	H-atom parameters constrained
*S* = 1.06	*w* = 1/[σ^2^(*F*_o_^2^) + (0.0725*P*)^2^ + 2.8897*P*] where *P* = (*F*_o_^2^ + 2*F*_c_^2^)/3
2987 reflections	(Δ/σ)_max_ < 0.001
235 parameters	Δρ_max_ = 0.28 e Å^−^^3^
0 restraints	Δρ_min_ = −0.32 e Å^−^^3^

### Special details

**Table:**

Geometry. All e.s.d.'s (except the e.s.d. in the dihedral angle between two l.s. planes) are estimated using the full covariance matrix. The cell e.s.d.'s are taken into account individually in the estimation of e.s.d.'s in distances, angles and torsion angles; correlations between e.s.d.'s in cell parameters are only used when they are defined by crystal symmetry. An approximate (isotropic) treatment of cell e.s.d.'s is used for estimating e.s.d.'s involving l.s. planes.
Refinement. Refinement of *F*^2^ against ALL reflections. The weighted *R*-factor *wR* and goodness of fit *S* are based on *F*^2^, conventional *R*-factors *R* are based on *F*, with *F* set to zero for negative *F*^2^. The threshold expression of *F*^2^ > σ(*F*^2^) is used only for calculating *R*-factors(gt) *etc*. and is not relevant to the choice of reflections for refinement. *R*-factors based on *F*^2^ are statistically about twice as large as those based on *F*, and *R*- factors based on ALL data will be even larger.

### Fractional atomic coordinates and isotropic or equivalent isotropic displacement parameters (Å^2^)

**Table:**

	*x*	*y*	*z*	*U*_iso_*/*U*_eq_	
Cl1	0.88491 (10)	0.2399 (2)	0.04201 (8)	0.0687 (4)	
O1	0.6161 (3)	0.7278 (4)	0.13219 (19)	0.0572 (9)	
O2	0.6094 (3)	0.8327 (5)	0.2452 (2)	0.0766 (11)	
O3	0.6181 (3)	0.3656 (5)	−0.10981 (19)	0.0663 (10)	
C2	0.6132 (4)	0.7045 (7)	0.2053 (3)	0.0559 (12)	
N3	0.6172 (3)	0.5233 (5)	0.2211 (2)	0.0519 (10)	
C4A	0.6211 (3)	0.4250 (6)	0.1587 (3)	0.0490 (11)	
C4	0.6275 (3)	0.2415 (6)	0.1450 (3)	0.0489 (11)	
H4A	0.6262	0.1538	0.1808	0.059*	
C5	0.6361 (3)	0.1930 (6)	0.0752 (3)	0.0506 (11)	
H5A	0.6400	0.0699	0.0640	0.061*	
C6	0.6391 (3)	0.3256 (6)	0.0204 (2)	0.0460 (11)	
C7	0.6294 (3)	0.5111 (6)	0.0358 (3)	0.0500 (11)	
H7A	0.6283	0.6010	0.0002	0.060*	
C7A	0.6219 (3)	0.5538 (6)	0.1039 (3)	0.0485 (11)	
C8	0.6265 (4)	0.4485 (8)	0.2965 (3)	0.0615 (13)	
H8A	0.6485	0.5460	0.3339	0.074*	
H8B	0.6821	0.3568	0.3101	0.074*	
C9	0.5278 (4)	0.3645 (6)	0.3019 (2)	0.0497 (11)	
C10	0.4392 (4)	0.4682 (7)	0.2953 (3)	0.0591 (13)	
H10A	0.4387	0.5917	0.2833	0.071*	
C11	0.3516 (4)	0.3913 (9)	0.3061 (3)	0.0722 (16)	
H11A	0.2927	0.4633	0.3021	0.087*	
C12	0.3507 (5)	0.2090 (9)	0.3226 (3)	0.0807 (17)	
H12A	0.2914	0.1570	0.3297	0.097*	
C13	0.4371 (6)	0.1042 (8)	0.3286 (3)	0.0806 (17)	
H13A	0.4360	−0.0200	0.3390	0.097*	
C14	0.5260 (5)	0.1800 (8)	0.3196 (3)	0.0698 (15)	
H14A	0.5852	0.1076	0.3253	0.084*	
C15	0.6501 (3)	0.2713 (6)	−0.0531 (2)	0.0451 (10)	
C16	0.7044 (4)	0.0938 (6)	−0.0560 (3)	0.0505 (11)	
C17	0.8106 (4)	0.0622 (7)	−0.0113 (3)	0.0518 (11)	
C18	0.8569 (5)	−0.1045 (8)	−0.0133 (3)	0.0679 (15)	
H18A	0.9267	−0.1260	0.0175	0.081*	
C19	0.8000 (6)	−0.2389 (8)	−0.0608 (4)	0.0817 (19)	
H19A	0.8317	−0.3515	−0.0617	0.098*	
C20	0.6969 (6)	−0.2108 (8)	−0.1073 (3)	0.0741 (17)	
H20A	0.6593	−0.3025	−0.1398	0.089*	
C21	0.6500 (5)	−0.0431 (7)	−0.1046 (3)	0.0653 (14)	
H21A	0.5806	−0.0224	−0.1362	0.078*	

### Atomic displacement parameters (Å^2^)

**Table:**

	*U*^11^	*U*^22^	*U*^33^	*U*^12^	*U*^13^	*U*^23^
Cl1	0.0556 (7)	0.0825 (10)	0.0667 (8)	−0.0025 (7)	0.0188 (6)	−0.0065 (7)
O1	0.070 (2)	0.0443 (19)	0.067 (2)	−0.0005 (15)	0.0354 (17)	−0.0036 (16)
O2	0.093 (3)	0.063 (2)	0.084 (3)	0.004 (2)	0.043 (2)	−0.019 (2)
O3	0.080 (2)	0.071 (2)	0.051 (2)	0.0075 (19)	0.0250 (17)	0.0077 (18)
C2	0.047 (3)	0.057 (3)	0.064 (3)	−0.001 (2)	0.019 (2)	−0.010 (3)
N3	0.057 (2)	0.050 (2)	0.052 (2)	0.0041 (18)	0.0221 (18)	−0.0039 (18)
C4A	0.044 (3)	0.049 (3)	0.058 (3)	0.002 (2)	0.022 (2)	0.000 (2)
C4	0.050 (3)	0.046 (3)	0.055 (3)	0.000 (2)	0.023 (2)	0.002 (2)
C5	0.052 (3)	0.049 (3)	0.057 (3)	−0.001 (2)	0.027 (2)	−0.002 (2)
C6	0.044 (2)	0.047 (3)	0.049 (3)	0.003 (2)	0.019 (2)	0.005 (2)
C7	0.050 (3)	0.049 (3)	0.056 (3)	−0.001 (2)	0.025 (2)	0.007 (2)
C7A	0.048 (3)	0.044 (3)	0.060 (3)	0.000 (2)	0.027 (2)	−0.002 (2)
C8	0.052 (3)	0.080 (4)	0.050 (3)	0.010 (3)	0.015 (2)	−0.009 (3)
C9	0.057 (3)	0.054 (3)	0.041 (2)	0.002 (2)	0.020 (2)	−0.005 (2)
C10	0.059 (3)	0.062 (3)	0.062 (3)	0.010 (2)	0.027 (2)	−0.002 (2)
C11	0.069 (4)	0.097 (5)	0.060 (3)	0.002 (3)	0.034 (3)	−0.013 (3)
C12	0.091 (5)	0.087 (5)	0.075 (4)	−0.022 (4)	0.041 (3)	−0.007 (3)
C13	0.115 (5)	0.059 (4)	0.077 (4)	−0.009 (4)	0.045 (4)	0.006 (3)
C14	0.096 (4)	0.059 (3)	0.063 (3)	0.013 (3)	0.038 (3)	0.000 (3)
C15	0.041 (2)	0.058 (3)	0.040 (2)	−0.001 (2)	0.0185 (18)	0.003 (2)
C16	0.056 (3)	0.052 (3)	0.053 (3)	−0.003 (2)	0.031 (2)	−0.001 (2)
C17	0.059 (3)	0.056 (3)	0.049 (3)	0.001 (2)	0.028 (2)	0.001 (2)
C18	0.082 (4)	0.064 (4)	0.071 (4)	0.016 (3)	0.044 (3)	0.009 (3)
C19	0.122 (6)	0.049 (3)	0.102 (5)	0.013 (4)	0.073 (5)	0.006 (3)
C20	0.119 (5)	0.060 (4)	0.066 (4)	−0.022 (3)	0.059 (4)	−0.015 (3)
C21	0.075 (4)	0.064 (4)	0.064 (3)	−0.015 (3)	0.033 (3)	−0.013 (3)

### Geometric parameters (Å, °)

**Table:**

Cl1—C17	1.732 (5)	C9—C14	1.392 (7)
O1—C2	1.384 (6)	C10—C11	1.376 (7)
O1—C7A	1.390 (5)	C10—H10A	0.9300
O2—C2	1.209 (6)	C11—C12	1.370 (8)
O3—C15	1.212 (5)	C11—H11A	0.9300
C2—N3	1.355 (6)	C12—C13	1.360 (9)
N3—C4A	1.381 (6)	C12—H12A	0.9300
N3—C8	1.472 (6)	C13—C14	1.374 (8)
C4A—C4	1.375 (6)	C13—H13A	0.9300
C4A—C7A	1.392 (6)	C14—H14A	0.9300
C4—C5	1.388 (6)	C15—C16	1.498 (6)
C4—H4A	0.9300	C16—C21	1.381 (7)
C5—C6	1.417 (6)	C16—C17	1.403 (6)
C5—H5A	0.9300	C17—C18	1.375 (7)
C6—C7	1.402 (6)	C18—C19	1.370 (8)
C6—C15	1.480 (6)	C18—H18A	0.9300
C7—C7A	1.341 (6)	C19—C20	1.375 (9)
C7—H7A	0.9300	C19—H19A	0.9300
C8—C9	1.491 (7)	C20—C21	1.386 (8)
C8—H8A	0.9700	C20—H20A	0.9300
C8—H8B	0.9700	C21—H21A	0.9300
C9—C10	1.378 (6)		
			
C2—O1—C7A	106.5 (4)	C11—C10—H10A	119.6
O2—C2—N3	129.2 (5)	C9—C10—H10A	119.6
O2—C2—O1	122.0 (5)	C12—C11—C10	120.2 (6)
N3—C2—O1	108.8 (4)	C12—C11—H11A	119.9
C2—N3—C4A	109.7 (4)	C10—C11—H11A	119.9
C2—N3—C8	123.8 (4)	C13—C12—C11	119.7 (6)
C4A—N3—C8	126.3 (4)	C13—C12—H12A	120.1
C4—C4A—N3	133.4 (4)	C11—C12—H12A	120.1
C4—C4A—C7A	120.5 (4)	C12—C13—C14	120.7 (6)
N3—C4A—C7A	106.0 (4)	C12—C13—H13A	119.7
C4A—C4—C5	117.0 (4)	C14—C13—H13A	119.7
C4A—C4—H4A	121.5	C13—C14—C9	120.4 (5)
C5—C4—H4A	121.5	C13—C14—H14A	119.8
C4—C5—C6	122.0 (4)	C9—C14—H14A	119.8
C4—C5—H5A	119.0	O3—C15—C6	122.4 (4)
C6—C5—H5A	119.0	O3—C15—C16	119.7 (4)
C7—C6—C5	119.3 (4)	C6—C15—C16	117.8 (4)
C7—C6—C15	119.6 (4)	C21—C16—C17	118.2 (5)
C5—C6—C15	121.1 (4)	C21—C16—C15	119.8 (4)
C7A—C7—C6	117.3 (4)	C17—C16—C15	121.9 (4)
C7A—C7—H7A	121.3	C18—C17—C16	120.4 (5)
C6—C7—H7A	121.3	C18—C17—Cl1	120.3 (4)
C7—C7A—O1	127.1 (4)	C16—C17—Cl1	119.2 (4)
C7—C7A—C4A	123.9 (4)	C19—C18—C17	119.8 (6)
O1—C7A—C4A	109.1 (4)	C19—C18—H18A	120.1
N3—C8—C9	115.2 (4)	C17—C18—H18A	120.1
N3—C8—H8A	108.5	C18—C19—C20	121.4 (5)
C9—C8—H8A	108.5	C18—C19—H19A	119.3
N3—C8—H8B	108.5	C20—C19—H19A	119.3
C9—C8—H8B	108.5	C19—C20—C21	118.7 (5)
H8A—C8—H8B	107.5	C19—C20—H20A	120.6
C10—C9—C14	118.1 (5)	C21—C20—H20A	120.6
C10—C9—C8	121.5 (5)	C16—C21—C20	121.4 (6)
C14—C9—C8	120.2 (5)	C16—C21—H21A	119.3
C11—C10—C9	120.9 (5)	C20—C21—H21A	119.3
			
C7A—O1—C2—O2	179.0 (4)	N3—C8—C9—C14	118.2 (5)
C7A—O1—C2—N3	0.1 (5)	C14—C9—C10—C11	0.2 (7)
O2—C2—N3—C4A	−179.8 (5)	C8—C9—C10—C11	−175.1 (4)
O1—C2—N3—C4A	−1.0 (5)	C9—C10—C11—C12	−0.9 (8)
O2—C2—N3—C8	−5.2 (8)	C10—C11—C12—C13	0.3 (9)
O1—C2—N3—C8	173.6 (4)	C11—C12—C13—C14	1.1 (9)
C2—N3—C4A—C4	178.8 (5)	C12—C13—C14—C9	−1.8 (9)
C8—N3—C4A—C4	4.4 (8)	C10—C9—C14—C13	1.2 (7)
C2—N3—C4A—C7A	1.5 (5)	C8—C9—C14—C13	176.5 (5)
C8—N3—C4A—C7A	−173.0 (4)	C7—C6—C15—O3	−24.5 (6)
N3—C4A—C4—C5	−176.3 (4)	C5—C6—C15—O3	154.4 (4)
C7A—C4A—C4—C5	0.8 (7)	C7—C6—C15—C16	154.7 (4)
C4A—C4—C5—C6	0.5 (6)	C5—C6—C15—C16	−26.4 (6)
C4—C5—C6—C7	−2.2 (6)	O3—C15—C16—C21	−61.6 (6)
C4—C5—C6—C15	178.9 (4)	C6—C15—C16—C21	119.1 (5)
C5—C6—C7—C7A	2.6 (6)	O3—C15—C16—C17	117.3 (5)
C15—C6—C7—C7A	−178.6 (4)	C6—C15—C16—C17	−62.0 (5)
C6—C7—C7A—O1	177.2 (4)	C21—C16—C17—C18	−3.3 (7)
C6—C7—C7A—C4A	−1.3 (7)	C15—C16—C17—C18	177.8 (4)
C2—O1—C7A—C7	−177.9 (4)	C21—C16—C17—Cl1	172.8 (4)
C2—O1—C7A—C4A	0.8 (5)	C15—C16—C17—Cl1	−6.1 (6)
C4—C4A—C7A—C7	−0.4 (7)	C16—C17—C18—C19	1.8 (7)
N3—C4A—C7A—C7	177.4 (4)	Cl1—C17—C18—C19	−174.2 (4)
C4—C4A—C7A—O1	−179.2 (4)	C17—C18—C19—C20	0.3 (8)
N3—C4A—C7A—O1	−1.4 (5)	C18—C19—C20—C21	−0.9 (8)
C2—N3—C8—C9	106.2 (5)	C17—C16—C21—C20	2.8 (7)
C4A—N3—C8—C9	−80.1 (6)	C15—C16—C21—C20	−178.3 (4)
N3—C8—C9—C10	−66.6 (6)	C19—C20—C21—C16	−0.7 (8)

### Hydrogen-bond geometry (Å, °)

**Table:**

Cg1 is the centroid of the C16–C21 ring.

**Table:**

*D*—H···*A*	*D*—H	H···*A*	*D*···*A*	*D*—H···*A*
C14—H14A···O2^i^	0.93	2.59	3.266 (8)	130
C20—H20A···O3^i^	0.93	2.59	3.269 (8)	130
C11—H11A···Cg1^ii^	0.93	2.92	3.474 (7)	119

Symmetry codes: (i) *x*, *y*−1, *z*; (ii) *x*−1/2, −*y*+1/2, *z*+1/2.
